# Supplementary material for: 12-O-tetradecanoylphorbol-13-acetate Reduces Activation of Hepatic Stellate Cells by Inhibiting the Hippo Pathway Transcriptional Coactivator YAP
Source: Cells. 2022 Dec 26;12(1):91. doi: 10.3390/cells12010091 (PMC9818550; doi:10.3390/cells12010091)
Supplement: Supplementary file 1 [file cells-12-00091-s001.zip › cells-2063842-supplementary.pdf]

Supplementary Material

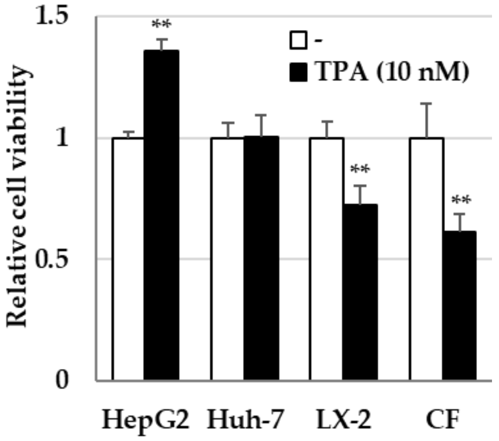

Figure S1. Effect of TPA on the proliferation of several cells. LX-2 and HepG2 cells were maintained in Dulbecco's modified Eagle medium (DMEM) supplemented with 3 % or 10 % fetal bovine serum (FBS), respectively. CFs were maintained in fibroblast growth medium (FGM)-3 with SingleQuots™ Supplements and Growth Factors (Lonza). Huh-7 cells were subcultured with Roswell Park Memorial Institute (RPMI) 1640 Medium (Gibco BRL) supplemented with 10 % FBS. Culture media were also supplemented with penicillin/streptomycin (Gibco BRL) for maintaining cells at 37 °C and 5 % CO_2. Cells were treated with 10 nM TPA for 2 days and then cell viability was analyzed using a MTT assay.

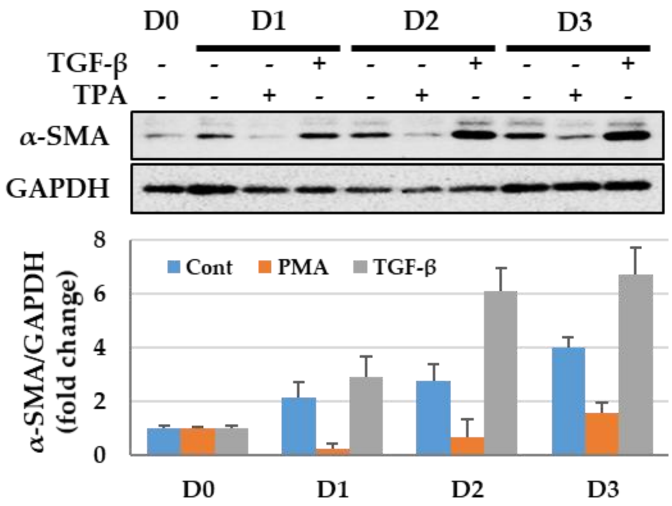

(a)

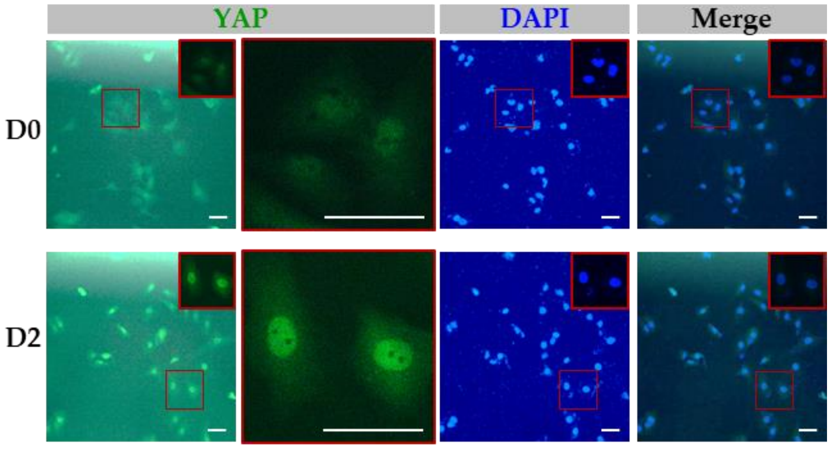

(b)

Figure S2. Expression of α-SMA and nuclear translocation of YAP in LX-2 cells. For the treatment of LX-2 cells with TPA (10 nM) or TGF-β for 2 days, we used DMEM media supplemented with 3 % FBS. (**a**) Expression of α-SMA in TPA- or TGF-β-treated LX-2 cells. In all groups, the expression of α-SMA was increased in a time-dependent manner, with the TGF-β-treated group showing the strongest increase, followed by the control group. The expression of α-SMA in the TPA-treated group was much lower than that in the control group. (**b**) Cellular distribution of YAP in LX-2 cells without TPA treatment at days 0 and 2. Scale bar, 20 μm.
